# An assessment of the multifactorial profile of steroid-metabolizing enzymes and steroid receptors in the eutopic endometrium during moderate to severe ovarian endometriosis

**DOI:** 10.1186/s12958-019-0553-0

**Published:** 2019-12-26

**Authors:** G. Anupa, Jai Bhagwan Sharma, Kallol K. Roy, Jayasree Sengupta, Debabrata Ghosh

**Affiliations:** 1 Department of Physiology, All India Institute of Medical Sciences, New Delhi, India; 2 Department of Obstetrics and Gynecology, All India Institute of Medical Sciences, New Delhi, India

**Keywords:** Aromatase, Endometriosis, Endometrium, Infertility, Menstrual cycle, Steroid receptors

## Abstract

**Background:**

Previous studies of expression profiles of major endometrial effectors of steroid physiology in endometriosis have yielded markedly conflicting conclusions, presumably because the relative effects of type of endometriosis, fertility history and menstrual cycle phases on the measured variables were not considered. In the present study, endometrial mRNA and protein levels of several effectors of steroid biosynthesis and action in patients with stage III-IV ovarian endometriosis (OE) with known fertility and menstrual cycle histories were compared with the levels in control endometrium to test this concept.

**Methods:**

Endometrial samples were collected from patients without endometriosis (*n* = 32) or OE stages III-IV (*n* = 52) with known fertility and cycle histories. qRT-PCR and immunoblotting experiments were performed to measure levels of NR5A1, STAR, CYP19A1, HSD17Bs, ESRs and PGR transcripts and proteins, respectively. Tissue concentrations of steroids (P4, T, E1 and E2) were measured using ELISAs.

**Results:**

The levels of expression of aromatase and ERβ were lower (*P* < 0.0001) and 17β-HSD1 (*P* < 0.0001) and PRA (*P* < 0.01) were higher in OE endometrium. Lower aromatase levels and higher 17β-HSD1 levels were detected in fertile (aromatase: *P* < 0.05; 17β-HSD1: *P* < 0.0001) and infertile (aromatase: *P* < 0.0001; 17β-HSD1: *P* < 0.0001) OE endometrium than in the matched control tissues. Both proliferative (PP) and secretory (SP) phase OE samples expressed aromatase (*P* < 0.0001) and ERβ (PP: *P* < 0.001; SP: *P* < 0.01) at lower levels and 17β-HSD1 (*P* < 0.0001) and PRA (PP: *P* < 0.01; SP: *P* < 0.0001) at higher levels than matched controls. Higher 17β-HSD1 (*P* < 0.01) and E2 (*P* < 0.05) levels and a lower (*P* < 0.01) PRB/PRA ratio was observed in infertile secretory phase OE endometrium than in control.

**Conclusions:**

We report that dysregulated expression of 17β-HSD1 and PGR resulting in hyperestrogenism and progesterone resistance during the secretory phase of the menstrual cycle, rather than an anomaly in aromatase expression, was the hallmark of eutopic endometrium from infertile OE patients. Furthermore, the results provide proof of concept that the fertility and menstrual cycle histories exerted relatively different effects on steroid physiology in the endometrium from OE patients compared with the control subjects.

## Introduction

Endometriosis is characterized by the presence of endometrial cells at ectopic loci and is often associated with chronic pelvic pain, dysmenorrhea, dyspareunia, dysuria, dyschesia and subfertility. It is a multifactorial disease that is widely prevalent among women of reproductive age. Retrograde menstruation followed by the adherence of stromal fibroblasts in the menstrual effluent is believed to be the pathophysiological mechanism underlying the onset of this disease [1]. Although retrograde menstruation occurs in 90% of women, only 1 in 10 women develop endometriosis, suggesting that an intrinsic anomaly in the eutopic endometrium of women with endometriosis may be one causal factor [2–5]. Endometriosis is generally postulated to be associated with steroid physiology in the target tissues [4, 5]. Increased activity of estrogen with or without progesterone resistance are suggested to be associated with this disease state [6]. The actions of estradiol-17β via its receptor (ER) are known to increase the proliferation of endometrial epithelial, stromal and endothelial cells in the eutopic endometrium of patients with endometriosis [7–9]. Additionally, a loss of progesterone receptivity and signaling *vis-à-vis* the suppression of progesterone receptor (PGR) activity in the endometrium and in ectopic lesions has been reported to be associated with endometriosis [10, 11]. Moreover, differential local metabolism of the major steroids, e.g., progesterone (P4), testosterone (T), estrone (E1) and estradiol-17β (E2), occurs in the eutopic endometrium and ectopic lesions during endometriosis in a menstrual phase-specific manner [12, 13]. In fact, several reports have described markedly conflicting results for the transcript and protein levels of the major steroid-synthesizing enzymes, steroidogenic co-factors, and the receptors for estrogen and progesterone in ovarian endometriosis (see Tables 1 and 2 for details). For example, marked differences in the level of aromatase activity have been observed in the endometrium of women with and without endometriosis. Noble et al. (1997) reported very low basal activity of aromatase in the eutopic endometrium of patients with endometriosis, as detected with a biochemical assay using 3H-androstenedione; however, aromatase activity in cultured endometrial stromal cells isolated from patients with endometriosis was increased by several fold in response to db-cAMP [14]. The expression of the CYP19A1 (aromatase) mRNA was found to be 14.5-fold higher in the mid-secretory phase, eutopic endometrium of infertile patients with mainly severe endometriosis of rectovaginal, peritoneal and ovarian subtypes compared with the control subjects. Additionally, endometrial stromal fibroblasts isolated from patients with endometriosis responded positively to PKA stimulation and displayed increased aromatase enzyme activity in vitro [16]. Huhtinen et al. (2012) similarly reported a low level of aromatase expression detected by using qRT-PCR in the mid-secretory eutopic endometrium of patients with a severe stage of endometriosis [12]. On the other hand, in several studies, aromatase activity was not detected in the eutopic endometria of women with and without endometriosis [15, 17, 18].

**Table 1:** Studies on factors regulating steroid synthesis in eutopic endometrium during ovarian endometriosis^a^

Reference [No.]	Type of endometriosis [sample type and size]	Major technique(s) employed	Salient observations
Noble et al., 1997 [14]	OE [CE (*n* = 7), EE (*n* = 2), EC (*n* = 4)]	Biochemical assay, qRTPCR	Low basal activity in EE but not reported for CE. Details of fertility status and phase of cycle was not provided.
Velasco et al., 2006 [15]	PE, OE [CE (*n* = 12), EE (*n* = 54), EC (*n* = 61)]	IHC	No immunopositive aromatase detected in CE and EE, while 61% of EC samples showed aromatase activity. Higher in secretory phase and in severe stage. No combinatorial analysis done. No details of fertility history was provided.
Aghajanova et al., 2009 [16]	PE, OE, DIE [CE (*n* = 13, EE (*n* = 29)]	qRTPCR, IHC	Aromatase mRNA expression was 14.5-fold upregulated, with no change in STAR, CYP11A1,HSD3B1, 2, CYP17A1 and HSD17B1, 2 in EE as compared to CE during mid-secretory phase of cycle. Fertility status was undefined.
Colette et al.,2009 [17]	OE, PE, RV [CE (*n* = 10), EE_EC (*n* = 56)]	IHC, WB in CE, EE and EC during both phases of menstrual cycle	Aromatase mRNA and protein were not detectable cycle. Perls’ stain^b^ positive siderophages were immunopositive for aromatase. Fertility history was not reported.
Delvoux et al., 2009 [18]	OE, DIE, SE [CE (*n* = 20), EE_EC (*n* = 14)]	Biochemical assay, HPLC	Aromatase activity was not detected in any tissue.No difference in reductive-oxidative activities of 17β-HSDs between CE and EE was observed. Details of menstrual cycle phase and fertility history were not reported.
Noel et al., 2011 [19]	PE, OE, DIE[CE (*n* = 16), EE_EC (*n* = 72)]	IHC. No details of stages of endometriosis, phases of menstrual cycle	No immunoreactivity of SF-1 was observed in EE. No details of stages of endometriosis, phases of cycle and fertility history was provided.
Huhtinen et al. 2012 [12]	PE, OE, DIE [CE (*n* = 11), EE (*n* = 17), EC (*n* = 18)]	qRTPCR	Generally, very low expression levels for mRNAs of CYP19A1 and HSD17B1, while high expression levels for HSD17B2, more during secretory phase, was detected in CE and EE with no difference between the groups. No details of stages of endometriosis and fertility history was provided.

^a^studies which did not (i) mention specifically the types of endometriosis, (ii) include CE as well as EE samples, (iii) include OE samples, were not selected

^b^Perls’ Prussian blue stain for ferritin. *CE* Control endometrium, *DIE* Deep infiltrating endometriosis, *EC* Ectopic lesion, *EE* Eutopic endometrium, *EE_EC* Autologous eutopic and ectopic tissues, *HPLC* High performance liquid chromatography, *IHC* Immunohistochemistry, *OE* Ovarian endometriosis, *PE* Peritoneal endometriosis, *qRTPCR* quantitative reverse transcriptase polymerase chain reaction, *RV* Rectovaginal endometriosis, *SE* Scar endometriosis, *WB* Western blotting

**Table 2:** Studies on estrogen receptor (ER) and progesterone receptor (PGR), and their subtypes in eutopic endometrium during ovarian endometriosis^a^

Reference[No.]	Type of endometriosis [sample type and size]	Major technique(s) used	Salient observations with remarks
Lessey et al., 1989 [20]	PE, OE, DIE [CE (*n* = 25), EE (*n* = 12), EC (*n* = 9)]	IHC	No difference was observed in the ER and PGR levels between CE and EE, as well as, between EE and EC. Analysis between CE and EE was not done based on phases of menstrual cycle despite tissue samples were collected during both cycle phases. Fertility history was not provided.
Burney et al., 2007 [10]	PE, OE [CE (*n* = 16), EE (*n* = 21)]	Microarray qRTPCR	2.3-fold downregulation of PGR mRNA was observed in EE compared to CE. Analysis between CE and EE was not done based on phases of menstrual cycle despite tissue samples were collected during both phases of menstrual cycle. Fertility history was not provided.
Bukulmez et al., 2008 [21]	PE, OE [CE (*n* = 8), EE (*n* = 12), EC (*n* = 14)]	qRTPCR, IHC, WB	Lower ESR1:ESR2 mRNA ratio was observed in CE and EE than EC along with higher immunopositivity for ERβ in EC. Lower PRA and PRB was observed in EC as compared to EE and CE. Analysis based on phases of menstrual cycle was not reported despite tissue samples were collected during both cycle phases. No information on stages of endometriosis and fertility history was provided.
Cavallini et al., 2011 [22]	OE [CE (*n* = 10), EE_EC (*n* = 10)]	qRTPCR, ELISA, IHC	Protein expression of ERα was higher in EE than CE and EC with similar expressions in mRNA profiles. Protein expression of ERβ was lower in EE than EC and CE. Higher PGR mRNA and immunopositivity was observedin EE than in EC. No data for stages of endometriosis, phases of menstrual cycle and fertility history was provided.
Huhtinen et al., 2012 [12]	PE, OE, DIE [CE (*n* = 15), EE (*n* = 37), EC (*n* = 41)]	qRTPCR	ESR1 mRNA expression was lower in both phases of menstrual cycle in EC than CE. ESR1 mRNA was found to be lower in secretory phase as compared to proliferative phase in EE. ESR2 mRNA was higher in EC (OE and DIE) than CE in both phases. No data for stages of endometriosis and fertility history was provided.

^a^studies which did not (i) mention specifically the types of endometriosis, (ii) include CE as well as EE samples, (iii) include OE samples were not selected. *CE* Control endometrium, *DIE* Deep infiltrating endometriosis, *EC* Ectopic lesion, *EE* Eutopic endometrium. *EE_EC* Autologous eutopic and ectopic tissues, *IHC* Immunohistochemistry, *OE* Ovarian endometriosis, *PE* Peritoneal endometriosis, *qRTPCR* quantitative real time PCR, *WB* Western blotting

**Fig. 1:**
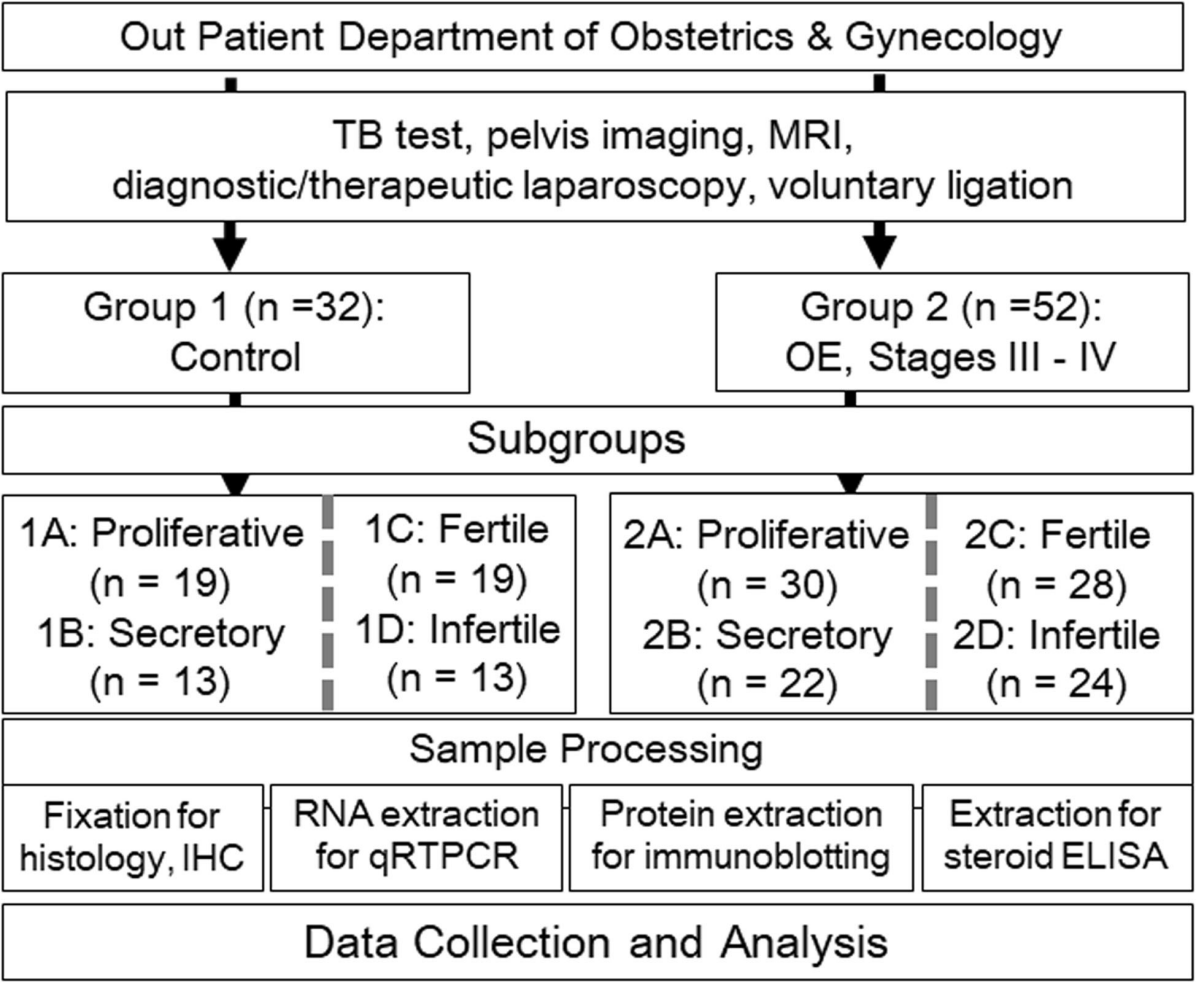
A schema showing a flow chart of the study design. In the present study, endometriosis-free patients (group 1) and patients with ovarian endometriosis (group 2) were recruited from the Department of Obstetrics and Gynaecology OPD, and the endometrial samples were collected according to the WERF EPHect guidelines and annotated according to fertility status and cycle phase. The transcript and protein expression profiles for NR5A1/SF-1, STAR/StAR, CYP19A1/aromatase, HSD17B1/17β-HSD1, HSD17B2/17β-HSD2, ESR1/ERα, ESR2/ERβ and PGR/PRA and PRB, as well as concentrations of progesterone (P4), testosterone (T), estrone (E1), estraiol-17β (E2) in the tissue samples, were determined using qRT-PCR, Western blot and steroid ELISAs, respectively. Data were analyzed and compared between the main groups (groups 1 and 2) and stratified according to the presence of stages III-IV ovarian endometriosis (OE). Data were also analyzed and compared between the subgroups and stratified according to the fertility status (groups 1A, 1B, 2A and 2B) and phase of the menstrual cycle (groups 1C, 1D, 2C and 2D), and the aforementioned subgroups were pooled for a combinatorial analysis

We hypothesized that marked inconsistencies among the observations of endometrial steroid physiology in previous studies might have resulted from the lack of a categorical consideration of the relative effects of fertility and menstrual histories on steroid hormone biosynthesis, metabolism and their receptors in the endometrium of patients with and without ovarian endometriosis (OE). The EPHect guidelines essentially highlight the necessity of developing a consensus on the standardization and harmonization of phenotypic surgical and clinical data and biological sample handling methods in endometriosis research [23, 24]. In the present study, endometrial samples obtained from thirty-two (32) control subjects and fifty-two (52) patients with moderate to severe (stages III-IV) OE who had a known fertility history and menstrual cycle phase registered in a tertiary hospital in New Delhi were examined to determine the intra-tissue concentrations of major sex steroid hormones (P4, T, E1 and E2) and the transcript and protein levels of steroid-synthesizing enzymes (CYP19A1/aromatase, HSD17B1/17β-HSD1, and HSD17B2/17β-HSD2), steroidogenic co-factors (NR5A1/SF-1 and STAR/StAR), and the receptors for estrogen (ESR1/ERα and ESR2/ERβ) and progesterone (PGR/PRA and PRB) to test this concept. To our knowledge, this study is the first to explore the relative effects of fertility history and menstrual cycle phases on the levels of effectors of steroid physiology in the eutopic endometrium during moderate and severe OE. A schema of the study design is shown in Fig. 1.

## Materials and methods

### Patient selection

Patients enrolled in the Department of Obstetrics and Gynecology of the All India Institute of Medical Sciences-Delhi for a surgical intervention for endometriosis, for evaluation at the Infertility Clinic or for family planning voluntarily participated in the study after understanding its purpose and providing written consent, according to the standard protocol. The study was approved by the Institutional Ethics Committee on the Use of Human Subjects (IEC/NP-3/2013; RP-08/04.03.2013) and conducted according to the Declaration of Helsinki Amendment 2013. Fertile patients and patients with primary infertility accompanied by stages III and IV ovarian endometriosis (OE) or no type of endometriosis were enrolled in the study as described elsewhere [25]. Exclusion criteria included the copresence of any other endocrinological disorder, cancer and uterine conditions, such as fibroids, adenomyosis, abnormal bleeding and tuberculosis, since these conditions might affect the results of the study, as described elsewhere [26, 27]. Only those patients who had not taken drugs such as contraceptives, GnRH analogues, aromatase inhibitors, danazol, dienogest or anti-tuberculosis therapy during the last 6 months and who had not undergone any previous laparoscopic surgery were included. Thirty-two (32) disease-free patients formed group 1 and fifty-two (52) patients diagnosed with stages III and IV ovarian endometriosis formed group 2. Table 3 provides a detailed description of the characteristics of the patients in the two groups.

**Table 3:** Patient details and sample distribution for each experiment

Group [N]	Subgroup (n)	Description	Age (Mean ± SD)	BMI (Mean ± SD)	Cycle day^a^ (Mean ± SD)	Number of samples used in each experiment		
						qRTPCR	WB	ELISA
Control (C) [28]	FPC (13)	Fertile Proliferative Control	25.3 ± 1.2	20.1 ± 1.1	8.2 ± 1.2	10	4	4
	IFPC (6)	Infertile^b^ Proliferative Control	26.8 ± 2.1	19.8 ± 1.2	10.5 ± 0.9	4	4	4
	FSC (6)	Fertile Secretory Control	32.4 ± 1.7	22.2 ± 1.3	24 ± 1.3	4	4	4
	IFSC (7)	Infertile^b^ Secretory Control	32.5 ± 2.7	22.4 ± 0.8	22 ± 1.8	4	4	4
Ovarian	FPU (17)	Fertile Proliferative Eutopic	31.9 ± 1.3	20.3 ± 0.9	9 ± 0.8	11	7	4
endometriosis	IFPU (13)	Infertile^b^ Proliferative Eutopic	27.2 ± 1.7	22.1 ± 1.3	11 ± 1.3	7	4	4
(OE) [29]	FSU (11)	Fertile Secretory Eutopic	34.4 ± 1.8	22.6 ± 1.7	22 ± 1.6	6	7	4
	IFSU (11)	Infertile^b^ Secretory Eutopic	19.6 ± 2.3	21.8 ± 1.1	23 ± 1.9	9	5	4

^a^cycle day of tissue collection

^b^primary infertility cases only. *BMI* Body mass index, *ELISA* Steroid ELISA, *qRTPCR* quantitative RTPCR, *WB* Western blotting

### Tissue processing

Proliferative and secretory phase endometrial samples obtained from upper uterine fundus were collected in cold phosphate-buffered saline (PBS, pH 7.4) using a Karmann cannula, and the samples were immediately washed with PBS, dissected into three parts and transported to the laboratory on ice. One portion was immediately pulverized in liquid nitrogen and stored at − 70 °C for Western blot experiments and steroid ELISAs, the second portion was incubated with Trizol for RNA extraction, which was stored at − 70 °C for qRT-PCR, and the third part was fixed with freshly prepared cold 4% (w/v) paraformaldehyde (Sigma-Aldrich Inc., St. Louis, MO, USA), processed and embedded in paraffin for the histological assessment of the endometrium.

### Quantitative RT-PCR (qRT-PCR)

The steady state expression levels of the transcripts for eight (8) target genes (NR5A1, STAR, CYP19A1, HSD17B1, HSD17B2, ESR1, ESR2 and PGR) were examined in isolated RNA samples with RIN scores of > 8.0 by using a real time RT-PCR platform (Bio-Rad CFX 96, Bio-Rad Laboratories, Hercules, CA, USA) and a protocol described elsewhere [27, 28]. Briefly, the RNA was reverse transcribed into cDNAs and then amplified using target gene-specific primers according to the manufacturer’s protocols (Thermo Fisher Scientific, Waltham, MA, USA). A reaction mixture was prepared in which 4 μL of reaction buffer, 1 μL of RiboLock RNase inhibitor (20 U/μL), 2 μL of 10 mM dNTP Mix and 1 μL of Revert Aid M-MuLV RT (200 U/μL) were added to the template (2 μg) and primer mix (1 μL forward and reverse primer) and heated to 42 °C for 60 min for amplification in a thermal cycler. The mixture was heated to 70 °C for 5 min to terminate the reaction and then cooled to 4 °C. The negative control was prepared with all reactants except the reverse transcriptase enzyme. A standard RNA for GAPDH provided with the kit was used at different concentrations to plot the standard curve used to determine the absolute levels of the transcripts of target genes [29]. The copy number was calculated from the expression levels using a standard formula (https://eu.idtdna.com/pages/education). Gene-specific forward and reverse primers were designed using the Beacon Designer v12.1 (Premier Biosoft, Palo Alto, CA, USA). The primers sequences are listed in Additional file 1: Table S1.

### Immunoblotting

Western immunoblotting (WB) experiments were performed for nine (9) target proteins (SF-1, StAR, aromatase, 17β-HSD1, 17β-HSD2, ERα, ERβ, PRA and PRB) to measure the relative levels of target proteins using standardized methods [28]. Briefly, the protein concentrations of each lysate were determined by using the Bradford assay, and 25 μg of proteins from each sample lysate and prestained molecular weight markers were separated by SDS-PAGE. WB was subsequently performed after proteins were transferred to nitrocellulose membranes using chemicals obtained from Bio-Rad (Hercules, CA, USA). The final visualization was achieved using Abcam Immunoperoxidase kits (Abcam, Cambridge, UK). The respective primary and secondary antibody controls were simultaneously incubated with the membranes to examine antibody specificity. The molecular weights were identified and semiquantitative analyses of the WB bands were performed using densitometry equipment (Pharos FX Molecular Imager) and the optimized densitometry analysis software (QuantityOne) from Bio-Rad (Hercules, CA, USA). For PRA and PRB, the intensities of respective bands were determined from the same runs, as described in a previous study [30]. The optical densities were measured from the log of inverse of transmittance for each target antigen, and the integrated optical densities were normalized to the total protein concentration determined by using the Bradford assay [28, 31]. Additional file 2: Table S2 provides a detailed description of the primary and secondary antibodies used for WB experiments.

### Steroid immunoassay

The concentrations of progesterone (P4), testosterone (T), estradiol-17β (E2) and estrone (E1) were measured in tissue lysates using commercially available ELISA kits obtained from Xema-Medica Co., Ltd. (Moscow, Russia) and Diametra Laboratories (Spello, Italy). For steroid ELISAs, the tissue lysates were prepared in Tris-EDTA buffer according to the manufacturer’s protocols. Briefly, tissue homogenates with an estimated protein concentration of 25 μg/ml were loaded in precoated wells of ELISA plates. The wells were then incubated with a conjugated antibody, washed to remove unbound and nonspecifically bound antibody, and then detected using TMB substrate-based detection methods. Tissue steroid concentrations are reported as pmol/mg of the total protein concentration measured by using the Bradford assay. Additional file 3: Table S3 provides the sensitivity, specificity, intra- and inter-assay coefficients of variances and percent recovery efficiency for each steroid estimated.

### Data analysis

Datasets for downstream analyses were categorized into the main groups (groups 1 and 2) according to the presence of OE, into subgroups according to the fertility (groups 1A, 1B, 2A and 2B), and menstrual (groups 1C, 1D, 2C and 2D) histories, and by pooling the abovementioned subgroups for a combinatorial analysis, as explained in the study design (Fig. 1) and group distributions (Table 3). The Kruskal-Wallis test followed by the Mann-Whitney U-test with the Bonferroni correction were used to calculate the statistical significance of the data with a non-Gaussian distribution obtained from the different experiments. Statistical analyses were performed using SPSS v 16.0 software (IBM Analytics, NY, US). In statistical inferences, *P* < 0.05 was considered significant.

## Results

### General characteristics

In the next sections, we report the results of the analyses of data used to investigate the effect of OE on the transcript and protein levels of steroid-synthesizing enzymes (CYP19A1/aromatase, HSD17B1/17β-HSD1, and HSD17B2/17β-HSD2), steroidogenic co-factors (NR5A1/SF-1 and STAR/StAR), and the receptors for progesterone (PGR/PRA and PRB) and estrogen (ESR1/ERα and ESR2/ERβ) and the intra-tissue concentrations of steroid hormones (P4, T, E1 and E2) in eutopic endometrial samples obtained from eighty-four (84) North Indian patients without and with endometriosis belonging to groups 1 (*n* = 32) and 2 (*n* = 52), respectively. We also examined the effects of the fertility status and the phases of menstrual cycle on the parameters examined. As shown in Table 3, the overall profiles of the patients were very similar, with no significant differences in the mean age, BMI and cycle days when the tissue was collected.

### Effect of endometriosis

**Fig. 2:**
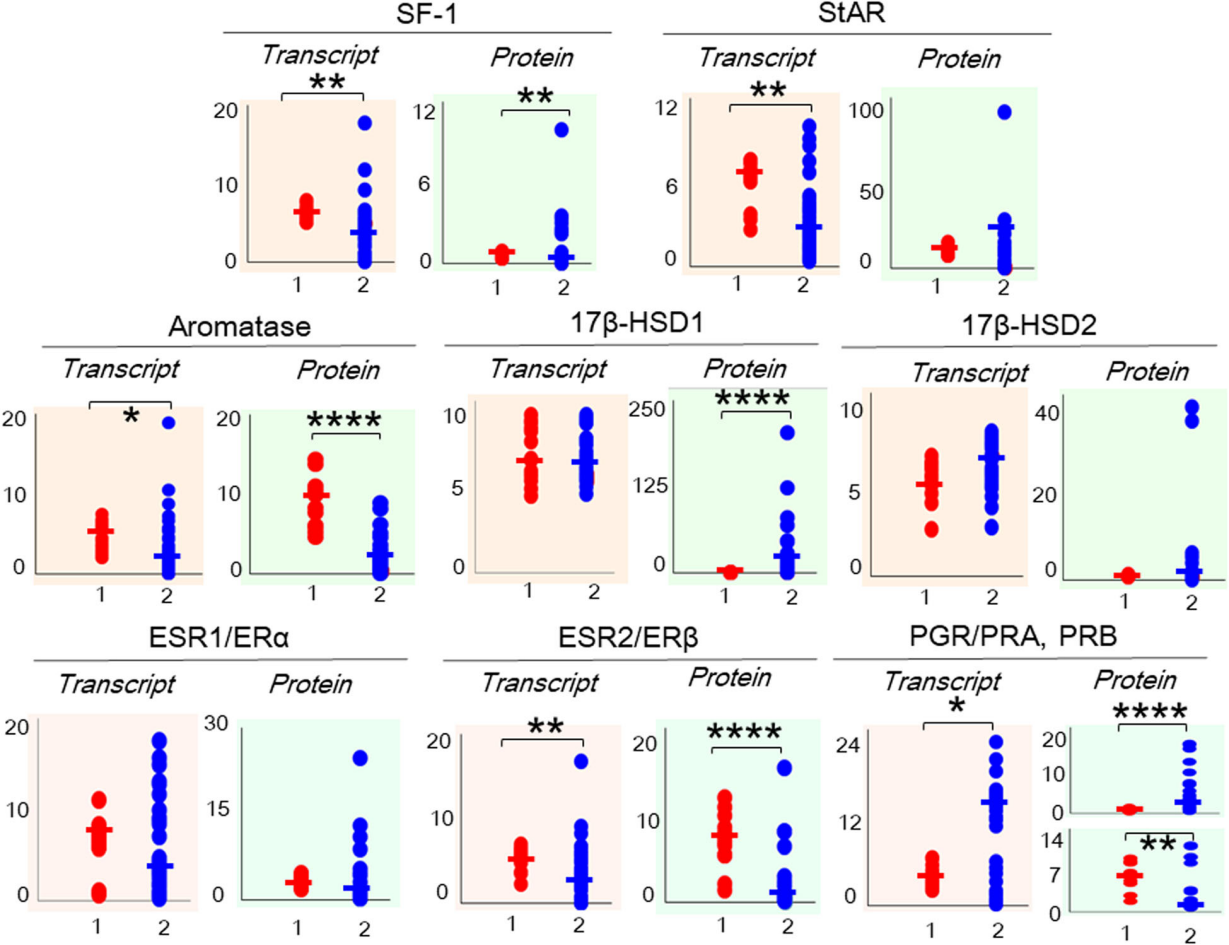
Transcript and protein levels in the control (group 1) and OE (group 2) groups. Trellis plots show log-transformed values for the transcript and protein expression data, along with the median values, for NR5A1/SF-1, STAR/StAR, CYP19A1/aromatase, HSD17B1/17β-HSD1, HSD17B2/17β-HSD2, ESR1/ERα, ESR2/ERβ, and PGR/PRA and PRB in endometrial samples obtained from patients without endometriosis (group 1), which are indicated by the *red dots*, and patients with OE (group 2), which are indicated by the *blue dots*. *P < 0.05, **P < 0.01, ***P < 0.001, and ****P < 0.0001

Figure 2 reports the levels of various transcripts and proteins examined in this study. The steady state levels of the NR5A1 (*P* < 0.01), STAR (*P* < 0.01), CYP19A1 (*P* < 0.05) and ESR2 (*P* < 0.01) transcripts were higher in samples from group 1 (control) than in samples from group 2 (OE). Among the factors showing higher transcript expression in group 1, significantly higher levels of the NR5A1 (i.e., SF-1) (*P* < 0.01), CYP19A1 (i.e., aromatase) (*P* < 0.0001) and ESR2 (i.e., ERβ; *P* < 0.0001) proteins were observed compared with group 2. Although the levels of the HSD17B1 and 2 transcripts and the 17β-HSD2 protein were not different between the groups, the 17β-HSD1 protein was expressed at lower levels (*P* < 0.0001) in the control (group 1) endometrium than in the eutopic endometrium from the OE group. Higher (*P* < 0.05) levels of the PGR and (*P* < 0.0001) PRA transcripts and lower (*P* < 0.01) levels of the PRB transcript were detected in group 2 (OE) than in group 1 (control). However, the steady state levels of the ESR1 and ERα transcripts and proteins did not show any differences between the two groups.

No significant differences in the steady state tissue concentrations of P4 [group 1: 263.9 (134.6–380.0) vs group 2: 111.9 (56.5–415.9); *P* = 0.87], T [group 1: 110.9 (46.7–162.0) vs group 2: 41.7 (16.5–166.0); *p* = 0.76], E1 [group 1: 22.1 (11.5–30.8) vs group 2: 8.0 (3.4–27.6); *P* = 0.10)], and E2 [group 1: 75.8 (23.4–157.9) vs group 2: 50.2 (10.9–118.0); *p* = 0.76] were observed between the two groups.

### Effect of the fertility status

**Fig. 3:**
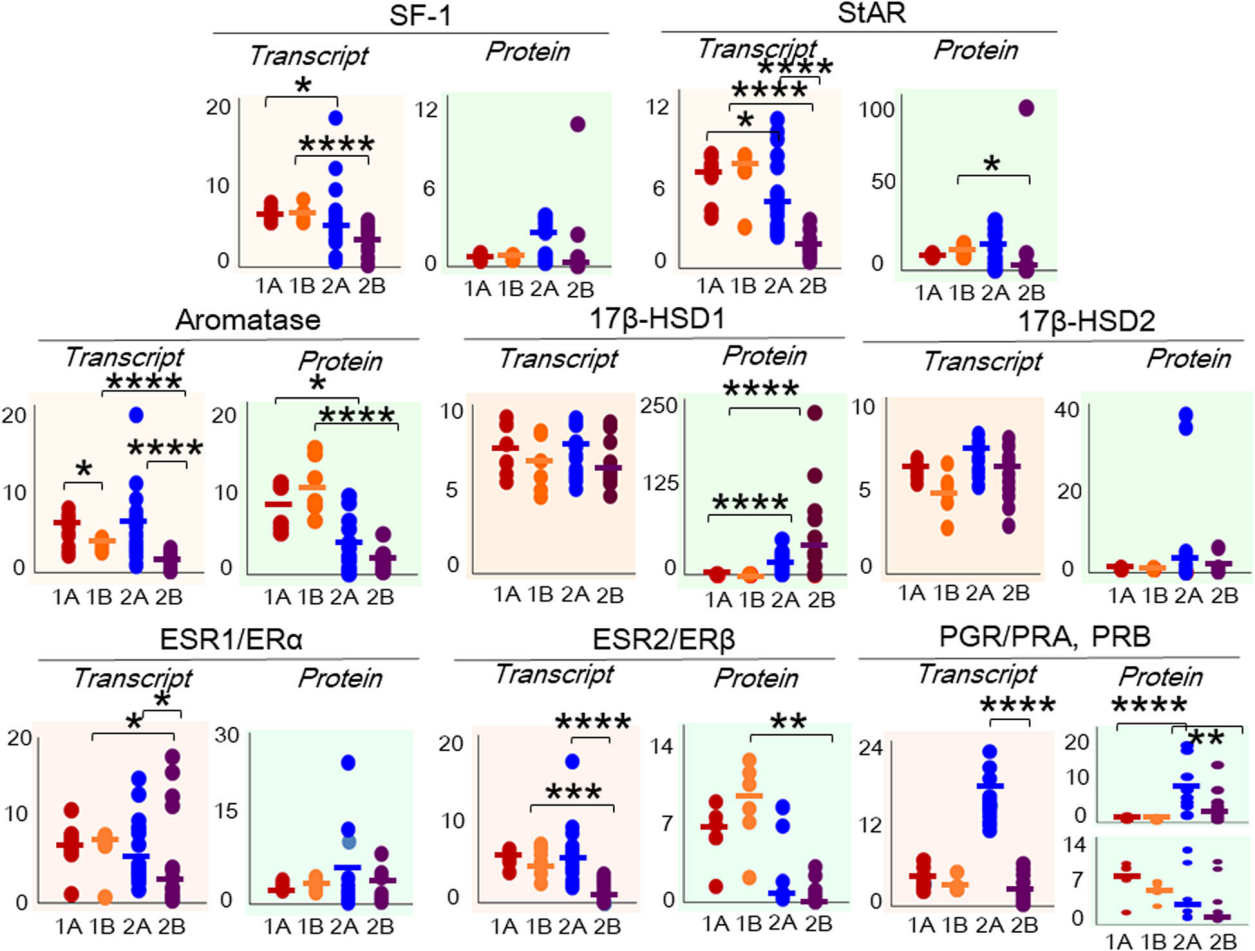
Effect of the fertility status on transcript and protein levels in the control (group 1) and OE (group 2) groups. Trellis plots show the log-transformed values for the transcript and protein expression data, along with the median values, for NR5A1/SF-1, STAR/StAR, CYP19A1/aromatase, HSD17B1/17β-HSD1, HSD17B2/17β-HSD2, ESR1/ERα, ESR2/ERβ, and PGR/PRA and PRB in endometrial samples obtained from control, fertile patients (group 1A), which are presented as *maroon dots*; control, infertile patients (group 1B), which are presented as *orange dots*; fertile patients with OE (group 2A), which are presented as *blue dots*; and infertile patients with OE (group 2B), which are presented as *violet dots*. *P < 0.05, **P < 0.01, ***P < 0.001, and ****P < 0.0001

Figure 3 shows the steady state transcript and protein levels for all the factors examined based on a supervised classification of the data in terms of the fertility status of the patients. Additional file 8: Figure S1 provides representative images of immunoblots from the different subgroups stratified by fertility status.

Intra-group comparisons between samples obtained from fertile and infertile patients were performed. A comparison of the levels of the target transcripts between samples obtained from control fertile (group 1A) and control infertile (group 1B) patients revealed higher (*P* < 0.05) CYP19A1 expression in the control fertile patients than in control infertile patients. However, no differences were observed in the protein levels of the other factors examined (SF-1, StAR, aromatase, 17β-HSDs, ERα, ERβ, PRA and PRB). In the comparison between eutopic fertile (group 2A) and eutopic infertile (group 2B) patients, markedly higher (*P* < 0.0001) levels of the STAR, CYP19A1, ESR2 and PGR transcripts and higher (*P* < 0.01) levels of the PRA protein were observed in fertile patients (group 2A) than in infertile patients (group 2B).

Inter-group comparisons between samples obtained from fertile and infertile patients were also performed. The fertile patients in group 1 (group 1A) presented higher (*P* < 0.05) levels of the NR5A1 and StAR transcripts than the eutopic fertile group (group 2A). Higher levels of the aromatase protein (*P* < 0.05) and lower levels of the 17β-HSD1 and PRA proteins (*P* < 0.0001) were detected in the control fertile patients (group 1A) than in eutopic fertile patients (group 2A). When samples from the control infertile group (group 1B) were compared with samples from the OE infertile group (group 2B), higher levels of the NR5A1 (*P* < 0.0001), STAR (*P* < 0.0001), CYP19A (*P* < 0.01), ESR1 (*P* < 0.05) and ESR2 (*P* < 0.001) transcripts were detected in group 1B than in group 2B. Higher levels of the StAR (*P* < 0.05), aromatase (*P* < 0.0001) and ERβ (*P* < 0.01) proteins were detected in the control infertile group (group 1B) than in the eutopic infertile group (group 2B). Lower levels of the 17β-HSD1 protein were observed (*P* < 0.00001) in group 1B than in group 2B. No changes were observed in the expression of HSD17B2 and 17β-HSD2 in the inter-group comparisons based on the fertility status.

A comparison of the steady state tissue concentrations of steroids revealed lower (*P* < 0.05) levels of testosterone (T) in the control fertile group (group 1A) than in the eutopic fertile group (group 2A), and higher (*P* < 0.05) levels of estrone (E1) were found in samples from the control infertile group (group 1B) than in the eutopic infertile group (group 2B). However, no significant difference was observed in the tissue concentrations of the other steroids studied among samples obtained from fertile and infertile patients with and without endometriosis.

In summary, similar trends of levels of the STAR/StAR, CYP19A1/aromatase, and HSD17B1/17β-HSD1 transcripts and proteins were observed in fertile and infertile patients from both groups. However, ESR1/ERα, ESR2/ERβ and PGR/PGA showed marked differences in fertile and infertile patients from the control and OE groups. Among the steroids examined, a lower tissue concentration of T was observed in the control fertile group (group 1A) than in the OE fertile group (group 2A), while a higher E1 concentration was detected in the control infertile group (group 1B) than in the OE infertile group (group 2B).

### Effect of phases of the menstrual cycle

**Fig. 4:**
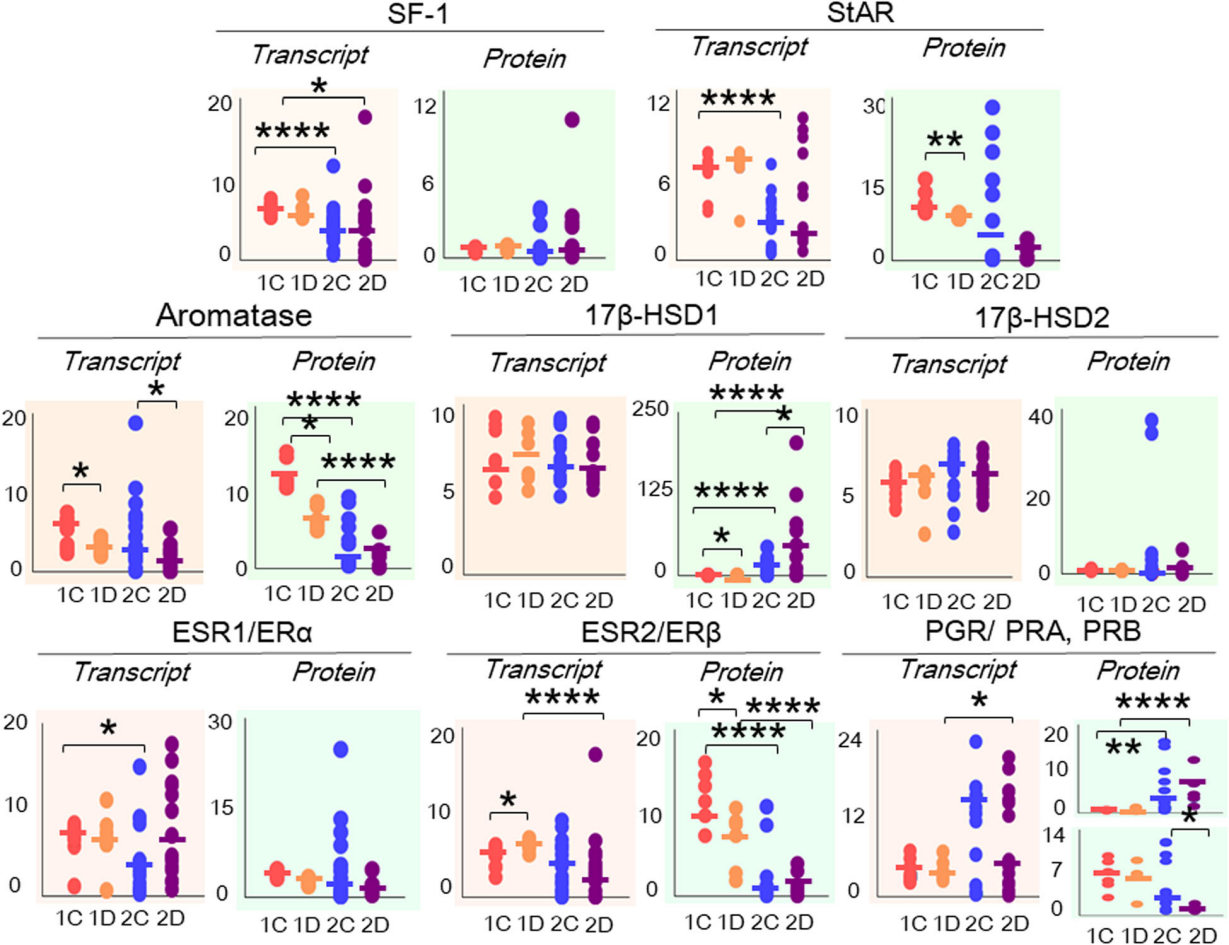
Effect of the menstrual cycle phase on transcript and protein levels in the control (group 1) and OE (group 2) groups. Trellis plots show log-transformed values for the transcript and protein expression data, along with the median values, for NR5A1/SF-1, STAR/StAR, CYP19A1/aromatase, HSD17B1/17β-HSD1, HSD17B2/17β-HSD2, ESR1/ERα, ESR2/ERβ, and PGR/PRA and PRB in the endometrium obtained from the control group in the proliferative phase (group 1C), which are presented as *pink dots*; the control group in the secretory phase (group 1D), which are presented as *orange dots*; the OE group in the proliferative phase (group 2C), which are presented as *blue dots*; and the OE group in the secretory phase (group 2D), which are presented as *purple dots*. *P < 0.05, **P < 0.01, ***P < 0.001, and ****P < 0.0001

Figure 4 shows the steady state transcript and protein levels of all the factors after the supervised classification of the data based on the menstrual cycle phase of the patients. Additional file 9: Figure S2 provides representative images of immunoblots from the different subgroups stratified according to the menstrual phase.

Intra-group comparisons between samples obtained during the proliferative and secretory phases were performed. A comparison between proliferative (group 1C) and secretory (group 1D) phases of group 1 (control) revealed higher levels (*P* < 0.05) of the CYP19A1 transcript and lower levels (*P* < 0.01) of the ESR2 transcript, along with higher levels of the StAR (*P* < 0.01), aromatase (*P* < 0.01), 17β-HSD1 (*P* < 0.05) and ESR2 (*P* < 0.05) proteins in the proliferative phase (group 1C) than in the secretory phase (group 1D). A comparison between the proliferative (group 2C) and secretory (group 2D) phases in group 2 (OE) revealed higher levels (*P* < 0.05) of the CYP19A1 transcript, lower levels (*P* < 0.05) of the 17β-HSD1 protein, and higher levels (*P* < 0.01) of the PRB protein in the proliferative phase (group 2C) than in the secretory phase in group 2D.

Inter-group comparisons of samples obtained during the proliferative phase and during secretory phase were also performed. As shown in Fig. 4, the comparison between samples obtained in the proliferative phase from group 1 (group 1C) and group 2 (group 2C) revealed higher expression of the NR5A1 (*P* < 0.0001), STAR (*P* < 0.0001) and ESR1 transcripts (*P* < 0.05), along with higher levels of the aromatase (*P* < 0.0001) and ERβ (*P* < 0.001) proteins and lower levels of the 17β-HSD1 (*P* < 0.0001) and PRA (*P* < 0.01) proteins in group 1C than in group 2C. The secretory phase samples from group 1 (group 1D) displayed higher expression of the NR5A1 (*P* < 0.05) and ESR2 (*P* < 0.0001) transcripts, lower expression of the PGR transcript (*P* < 0.05), higher levels of the aromatase (*P* < 0.0001), ERβ (*P* < 0.01) and PRB (*P* < 0.0001) proteins, and lower levels (*P* < 0.0001) of the 17β-HSD1 and PRA proteins than to secretory phase samples (group 2D). No change was observed in the expression of HSD17B2 and 17β-HSD2 in the inter-group comparisons based on the phase of menstrual cycle.

No significant differences were observed in the tissue concentrations of P4 [group 1C: 167.3 (87.2–380.0), group 1D: 184.9 (84.1–331.9), group 2C: 110.9 (56.5–184.0), group 2D: 253.0 (101.3–415.0); *P* = 0.45)], T [group 1C: 63.1 (27.2–161.8), group 1D: 65.9 (46.6–142.8), group 2C: 44.4 (16.5–85.4), group 2D: 113.4 (49.9–165.8); *P* = 0.51], E1 [group 1C: 12.3 (9.2–40.3), group 1D: 13.5 (8.1–28.8), group 2C: 6.8 (3.4–16.2), group 2D: 18.3 (10.2–36.9); *P* = 0.12], and E2 [group 1C: 57.6 (13.2–157.9), group 1D: 25.5 (6.7–69.0), group 2C: 23.1 (10.9–50.2), group 2D: 81.5 (23.1–118.0); *P* = 0.64].

In summary, the expression of the NR5A1 (SF-1), CYP19A1 (aromatase), HSD17B1 (17β-HSD1), and ESR2 (ERβ) transcripts and proteins showed similar trends in the proliferative phase and secretory phase of the menstrual cycle in the control and OE groups. However, marked differences were noted in PGR (PRA and PRB) expression between the two groups. Significant differences in the steady state concentrations of the steroids analyzed were not observed in the intra-group and inter-group comparisons of the proliferative phase and secretory phase samples from the control and OE groups.

### Combinatorial effects of the fertility status and menstrual cycle phase

**Fig. 5:**
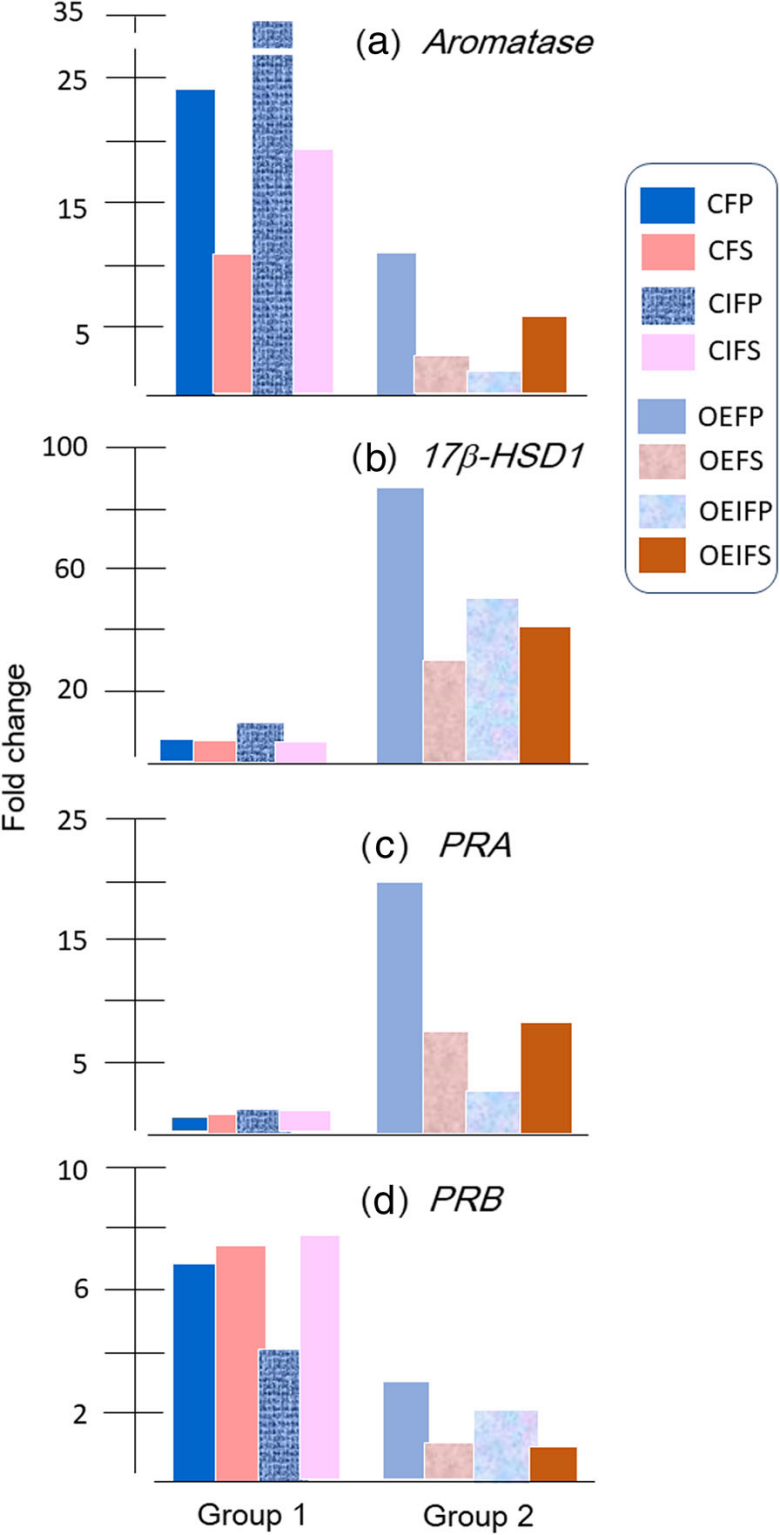
Fold-change profile of immunopositive aromatase (**a**), 17β-HSD1 (**b**), PRA (**c**) and PRB (**d**) proteins in different subgroups as shown in the legend. The minimum median value was taken as 100%. Relatively higher expression of 17β-HSD1 and lower expression aromatase in endometrium from OE group is suggestive of hyperestrogenism in OE due to higher 17β-HSD1 rather than aromatase. Also, higher expression of PRA along with lower expression PRB in endometrium from OE group is suggestive of relative lack of progesterone mediated secretory preparation in endometrium during OE. C, control; F, fertile; IF, infertile; OE. Ovarian endometriosis; P, proliferative phase; S, secretory phase. For details, see Additional files 4, 5, 6 and 7: Tables S4–S7

Steady state transcript and protein levels of all factors and the intra-tissue concentrations of steroids were also examined after the supervised classification of the data based on the fertility and the menstrual cycle histories of the patients (for details see Additional files 4, 5, 6: Tables S4-S6). The parameters displaying marked changes are summarized in Fig. 5.

A comparative analysis between the fertile proliferative phase subgroup in group 1 (control) and fertile proliferative phase subgroup in group 2 (OE) revealed higher levels (*P* < 0.05) of the aromatase protein in group 1 than in group 2. However, the transcript and protein levels of the other factors studied remained unchanged between these two groups. A comparison between infertile secretory endometrial samples obtained from group 1 (control) and group 2 (OE) revealed higher 17β-HSD1 (*P* < 0.01) and PRA (*P* < 0.05) levels along with lower levels of the PRB (*P* < 0.01) protein in samples from group 2. Among patients with OE (group 2), higher levels of the PGR transcript (*P* < 0.01) and aromatase protein (*P* < 0.05) were detected in the infertile secretory phase endometrium than in infertile proliferative phase endometrium. Steady state measurements of the intra-tissue concentrations of steroids after the supervised classification of the dataset based on the fertility status and the menstrual cycle phase of the patients did not reveal noticeable changes in the concentrations of the steroid hormones studied, with the exception of E2 levels, which were higher (*P* < 0.05) in the secretory phase endometrial samples from the infertile patients in group 2 (OE) than in the fertile group.

In summary, the eutopic endometrium of infertile patients with OE exhibited markedly higher (*P* < 0.01) 17β-HSD1 levels, higher (*P* < 0.05) tissue E2 levels, and a lower (*P* < 0.01) PRB:PRA ratio than the control, infertile, secretory phase endometrium during the secretory phase.

## Discussion

In general, endometriosis is postulated to be associated with hyperestrogenism and progesterone resistance [3, 5, 32–37]. However, as evident from the data presented in Tables 1 and 2, marked incongruences in the reported profiles of transcripts and proteins for major steroid-synthesizing enzymes, steroidogenic co-factors, and the receptors for estrogen and progesterone have been observed in the eutopic endometrium obtained from patients with OE compared to the disease-free endometrium. We hypothesized that these discrepancies in the findings of previously reported studies might have been attributed to various insufficiencies in the patient grouping and data processing methods, such as an analysis of pooled data from patients with different stages of endometriosis, from samples collected from fertile and infertile patients, and from samples collected during the different phases of menstrual cycle. All these variables are known to affect the final observations of the expression and activities of steroid-metabolizing enzymes, co-factors, and steroid receptors in the endometrium [20, 38–40]. In an attempt to circumvent these limitations, we adopted a method of clear annotation and processing of samples to perform a comparative multiparameter assessment of factors related to estrogen and progesterone turnover and their actions in the eutopic endometrium of patients with OE and the disease-free endometrium and to study the relative effects of the fertility status and phases of menstrual cycle on these parameters. The present study is the first to substantiate the concept that fertility and menstrual cycle histories differentially affect the endometrial steroid physiology in patients with OE compared to patients with a disease-free endometrium.

In contrast to previous reports describing higher aromatase levels in the eutopic endometria of patients with endometriosis than in the endometrium from disease-free women, as detected by using RT-PCR and immunochemistry [21, 41], we report significantly lower levels of the CYP19A1/aromatase mRNA and protein in the eutopic endometrium of women with diagnosed OE in a menstrual phase-specific manner, regardless of their fertility status. This lack of concordance between previous reports and the present study might have several explanations, as discussed below.

1. The samples analyzed in the many of the previous studies were obtained from patients with different types of endometriosis [12, 17–19, 23] or from patients with extraovarian endometriosis [42, 43]. However, in the present study, samples were obtained from patients with stages III-IV OE and provided highly specific information about aromatase expression in the endometrium of patients with and without OE.
2. Another potential explanation is the differences in methodologies adopted in previous studies. For example, Kitawaki et al. (1997) employed Southern blot experiments [41], and Bukulmez et al. (2008) assessed the relative expression of untranslated exon IIa to estimate the expression of CYP19A1 transcripts [21]. In this study, we have performed absolute quantification of the copy numbers of the CYP19A1 transcript using the best primers designed by Beacon Designer, which were free of primer-dimer and secondary structures, in qRT-PCR.
3. Ethnic differences might have explained the observed differences. Single nucleotide polymorphisms leading to altered mRNA splicing in the intronic regions of CYP19A1 result in genotypic and allelic variability among populations of women of European and African ancestry [44]. Four different ancestries with wide genetic diversity exist in the Indian subcontinent [45], and differences in single nucleotide polymorphisms in CYP19A1 between women hailing from North India [46] and South India [47] have been observed, which may be associated with differential effects on steroid biochemical phenotypes and altered disease susceptibilities [48–50].
4. Finally, as described above, most of the previous reports failed to create segregated bins in the data analysis pipeline based on type of endometriosis, severity stage, fertility and menstrual cycle histories, resulting in significant noise in the data mining process. This limitation is now well-acknowledged to frequently yield confusing results. In fact, the WERF EPHect guidelines recommend the adoption of standardized methods for clear annotation, sampling and data mining based on optimized and valid data segregation approaches to avoid the expected high noise in the results [23, 24].

### Higher 17β-HSD1, but not aromatase, expression is associated with hyperestrogenism in the endometrium during ovarian endometriosis

Despite marked intra-group variations, the observed higher steady state levels of the NR5A1 and CYP19A1 transcripts and CYP19A1 (aromatase) protein in samples from the control group compared with samples from the OE group, particularly samples from fertile patients, did not corroborate well with a previous report showing that the normal endometrium lacked the ability to synthesize estrogen from androgens due to the absence of StAR and aromatase [12, 14, 17, 34, 37]. However, Tseng et al. (1982) previously reported aromatase activity in the disease-free human endometrium [28]. The expression of 17β-HSD1, which catalyzes the NAD(P)H-dependent reduction of estrone into estradiol [40], was lower in the control endometrium than in OE samples, particularly samples from patients with confirmed fertility. In this connection, the observations of marginally but consistently lower tissue testosterone concentrations in the endometrium from control, fertile patients than in samples obtained from fertile patients with OE, along with a slightly higher level of estrone in samples from the control infertile group than in samples from the eutopic infertile group, might reflect a stochastic mechanism with systems bias in steroid processing in the respective tissues [51]. The physiological importance of marginal changes in the steroid levels in the presence of a robust mover has been addressed in a previous study [52].

As expected, menstrual cycle phase-specific variations in the transcript and protein levels of various enzymes and co-factors, including SF-1, StAR, aromatase and 17β-HSDs, were detected. Notably, relatively higher intra-tissue concentrations of E2 and 17β-HSD1 were observed in infertile patients with endometriosis during the secretory phase. As mentioned above, high 17β-HSD1 levels tend to increase the E2 output by about 4-fold in the tissue [29, 40]. Furthermore, the possibility that the local E2 profile was influenced by aromatase-independent pathways involving the production of E1 from estrone sulfate or dehydroandrostenedione (DHEA) from DHEAS, and the conversion of E1 to E2 and DHEA to androstenediol by 17β-HSD1, as observed in hormone responsive primary breast cancer [53] and endometrial cancer [54], must be examined. Notably, higher expression of steroid sulfatase (STS) was observed in stromal cells from the eutopic endometrium of patients with endometriosis [55].

Infertility is prevalent among patients with OE [56–58]. A plausible hypothesis is that hyperestrogenism in the endometrium during the secretory phase in patients with endometriosis is a likely cause of infertility. Cellular aberrations described in the eutopic endometrium of endometriosis have been observed in the stratum functionalis in the secretory phase, where a persistence of proliferative activity is detected [59–61]. Eutopic stromal cells from patients with OE show a reduced capacity for decidualization that affects their capacity for proliferation and survival in the ectopic environment [16, 62]. Elevated E2 levels in the eutopic tissue from patients with OE may play a role in disease progression by upregulating the tissue expression of ß-catenin [63], which regulates cell adhesion and migration and functions as a transcription factor regulating endometrial differentiation via the Wnt signaling pathway [64].

Based on the results obtained in the present study, we concluded that eutopic endometrium of patients with OE displayed hyperestrogenism primarily due to dysregulated 17β-HSD1, particularly in the secretory phase of the menstrual cycle, which may be a cause of the higher rate of implantation failure in this group [3, 4]. Furthermore, Delvoux et al. (2014) revealed that 17β-HSD1 was a major driving factor for the imbalance in estrogen turnover in endometriotic lesions and suggested that the inhibition of this enzyme might be a potential future treatment strategy for restoring the correct metabolic balance targeted to patients with endometriosis presenting increased local 17β-HSD1 enzyme activity [65]. The scenario may be different in patients with deep infiltrating endometriosis (DIE), which is characterized by the suppression of 17β-HSDs 2 and 4 along with increased expression of aromatase and 17β-HSD1 [66]. Further studies are warranted to examine these hypotheses.

### Dysregulated endometrial progesterone receptor in ovarian endometriosis and infertility

Higher levels of PRA and lower levels of the ERβ and PRB proteins, along with a higher level of the PGR transcript, were detected in the OE group than in the disease-free control group. For estrogen, two structurally related ER subtypes, ERα and ERβ – which are products of two separate genes – signal when complexed with E2. Although the involvement of the ER subtypes (ERα and ERβ) in the progression of endometriosis is not clear [67, 68], the results obtained from the present study of North Indian women concur with the findings reported by Zhang et al. (2018), who also did not observe any change in the levels of the wild type ERα mRNA in a population of fertile Chinese women with and without endometriosis [69].

In the coordinated receptor model for estrogen-mediated signaling in human endometrium proposed by Miller and associates (2018), the ERa66 variant is responsible for inducing receptor-mediated signaling cascades to promote cell proliferation along with the activation of a negative regulatory mechanism mediated by ERβ and Era46 to maintain homeostasis in the presence of hormone transients [70]. In contrast to ERα, the low levels of the ESR2 mRNA and ERβ protein in the eutopic endometrium of women with moderate to severe OE compared with healthy women observed in the present study are consistent with the low ERβ levels reported in cells of the eutopic endometrium from patients with endometriosis, which were positively correlated with increased telomerase expression that indicated a persistently greater proliferative phenotype [71, 72]. While we were unable to detect any marked changes in the ERα:ERβ ratio in the endometria of women with and without OE, a trend toward higher expression was noted in women with OE. An analysis of the classical paradigm based on the ligand binding-dimerization-transcription-proliferation of ER subtypes in endometrial cells of normal and OE tissues appears to be warranted to resolve the issue of the marginal shift in the ERα:ERβ ratio in the OE endometrium [73].

Regarding the progesterone receptor, our observations were consistent with a previous report showing a higher PRA:PRB ratio due to aberrant overexpression of PRA in the eutopic endometrium during OE [74, 75]. Progesterone responsiveness in the endometrium is mediated by the coordinated actions of two receptor isoforms, PRA and PRB, which are transcribed from two different promoters of the single PR gene. One hundred sixty-four amino acids are missing from the amino terminus of PRA compared to PRB [76]. Progesterone action in uterine tissues is qualitatively and quantitatively determined by the relative levels and transcriptional activities of PRA and PRB [77–79]. Human PRB is known to function as an activator of progesterone-responsive genes, while PRA is transcriptionally inactive and additionally functions as a strong transdominant repressor of PRB and ER transcriptional activity [76–79]. In the normal endometrium, the PR isoforms are evenly distributed in the proliferative phase, while PRB is the predominant isoform in nuclear foci in the secretory phase, resulting in a higher PRB:PRA ratio [80]. The results of the present study corroborate the levels of PRA and PRB based on Western immunoblotting of the control, disease-free endometrium, while the higher PRA:PRB ratio observed in samples from patients with moderate to severe OE may be associated with the subsequent repression of PRB activity in the secretory phase of infertile patients. In patients with moderate to severe OE, the environment of the eutopic endometrium appears to undergo a loss of the normal luteal-phase dominance of progesterone with a higher ratio of PRA:PRB, resulting in progesterone resistance and estrogen dominance [81]. In an elegant study, Barragan et al. (2016) observed that human endometrial fibroblasts display progesterone resistance in the endometrial niche in endometriosis [82]. This dysregulated progesterone action notably results in hyperplastic noise in the endometrium [83]. Progesterone action in the secretory phase endometrium is sine qua non for promoting endometrial differentiation and receptivity for embryo implantation in primates [84–86]. Thus, as observed in the present study, dysregulated P receptivity in infertile patients with OE might be a mechanism underlying the anomalous endometrial gene expression observed in women with repeated implantation failure and infertility [87–89].

### Limitations and strengths of the study

The present study has a major limitation due to the markedly dispersed data points for most of the parameters. This dispersion, combined with supervised factorial supra-binning of data, resulted in a reduction in the number of data points for each subgroup. Nevertheless, our protocol of serially binning the data into groups and subgroups provided the proof of an original concept that differential regulatory homeodynamics of steroids occur in the endometrium, depending on the phases of the menstrual cycle, fertility history and presence of endometriosis.

Furthermore, we did not observe a good correlation between the transcript and corresponding protein levels in the present study, with the exception of the correlations between the levels of the ESR1 and ERα protein and between the levels of the StAR transcript and protein (Additional file 7: Table S7). Good correlations between mRNA and protein levels enable protein levels to be predicted from mRNA levels, which are able to be collected more accurately and easily in a high-throughput manner [90, 91]. Since the mRNA is eventually translated into protein, a reasonable assumption is that some correlation should exist between the mRNA and protein levels. The steady state levels of various mRNAs represent a profile of the related genomic expression and provides useful values in a broad range of applications, including the diagnosis and classification of disease, but these results are only correlative and not causative [92, 93]. On other hand, the concentration of proteins and their interactions reflect causative pathways in the cell [91, 94]. Thus, the quantification of both of these molecular populations is not an exercise in redundancy; measurements of mRNA and protein levels are complementary, and both are necessary to obtain a complete understanding of a physiological state, even if an overt correlation does not exist between these two sets of data, as observed in the present study [95]. At least three reasons presumably explain the poor correlations between the mRNA and protein levels, which may not be mutually exclusive [95]. First, many complex and dynamic posttranscriptional mechanisms are involved in the ultimate translation of the mRNA into a protein, and our understanding of these processes is grossly insufficient. Second, proteins generally differ substantially in their half-lives in situ. Third, a significant signal-noise ratio and error exist in both protein and mRNA experiments, which are also not hyperstatic modules. All these biological properties are dynamic and depend on the biochemical nuances of the attractor properties of the homeodynamics of particular physiological and pathophysiological states [96].

Thus, based on our observed results revealing a marked lack of correlation and correspondence in the mRNA and protein levels examined in the endometrium obtained from fertile and infertile patients with or without OE during different phases of cycle, we conjecture that differential regulatory homeodynamics of the steroids occur in the human endometrium, depending on its ecological succession with the phase of menstrual cycle, fertility history and the presence of endometriosis [86, 97].

Finally, we report for the first time that there exists lower levels of the CYP19A1/aromatase mRNA and protein in the eutopic endometrium of women with diagnosed OE in a menstrual phase-specific manner, regardless of their fertility status. Thus, we conclude that dysregulated 17β-HSD1 expression and alterations in the PRA:PRB ratio resulting in hyperestrogenism and progesterone resistance during the secretory phase of the menstrual cycle, rather than an anomaly in aromatase expression, were the hallmarks of the eutopic endometrium of infertile patients with OE. Moreover, our results provide proof of concept for the different effects of the fertility history and menstrual cycle phases on steroid physiology in the endometrium of patients with moderate to severe OE compared with control subjects.

## Supplementary information

**Additional file 1:** **Table S1.** List of primers for qRTPCR.

**Additional file 2: Table S2.** List of primary antibodies used in Western immunoblotting.

**Additional file 3:** **Table S3**. Sensitivity, specificity, intra- and inter-assay coefficients of variances (CV) and per cent recovery efficiency of endometrial tissue steroids estimated.

**Additional file 4:** **Table S4.** Endometrial transcript and protein expression of factors involved in steroid biosynthesis in patients with and without endometriosis.

**Additional file 5:** **Table S5.** Endometrial transcript and protein expression of estrogen and progesterone receptors in patients with and without endometriosis.

**Additional file 6:** **Table S6.** Tissue steroid hormone concentration (pmol/mg protein) in endometrium of patients with and without endometriosis.

**Additional file 7:** **Table S7.** Correlation between transcripts and protein levels.

**Additional file 8:** **Figure S1.** Representative images of Western blots showing the effect of the fertility history on the levels of the SF-1, StAR, aromatase, 17β-HSD1, 17β-HSD2, ERα, ERβ, PRA and PRB proteins in endometrial samples from the control, fertile (C,F; group 1A); control, infertile (C,IF; group 1B); OE, fertile (OE,F; group 2A); and OE, infertile (OE,IF; group 2B) groups. Tissue lysates of samples from these groups (25 μg of proteins, concentrations were determined by using the Bradford assay) were subjected to electrophoretic separation followed by immunoblot analysis. The relative optical densities were measured by performing an integrated image analysis and normalizing the value to the μg of total protein.

**Additional file 9:** **Figure S2.** Representative images of Western blots showing the effect of the menstrual cycle phase on the levels of the SF-1, StAR, aromatase, 17β-HSD1, 17β-HSD2, ERα, Erβ, PRA and PRB proteins in endometrial samples from the control, proliferative (C,P; group 1C); control, secretory (C,S; group 1D); OE, proliferative (OE,P; group 2C); and OE, secretory (OE,S; group 2D) groups. Tissue lysates of samples from these groups (25 μg of protein, concentrations were determined by using the Bradford assay) were subjected to electrophoretic separation followed by immunoblot analysis. The relative optical densities were measured by performing an integrated image analysis and normalizing the values to the μg of total protein.

## Data Availability

All the data and residual materials are well preserved and hence available with the group.
