# Supplementary material for: An assessment of the multifactorial profile of steroid-metabolizing enzymes and steroid receptors in the eutopic endometrium during moderate to severe ovarian endometriosis
Source: Reprod Biol Endocrinol. 2019 Dec 26;17:111. doi: 10.1186/s12958-019-0553-0 (PMC6933937; doi:10.1186/s12958-019-0553-0)
Supplement: Supplementary file 1 — Additional file 1: Table S1. List of primers for qRTPCR. [file 12958_2019_553_MOESM1_ESM.docx]

Additional file 1: Table S1 List of primers for qRTPCR

_____________________________________________________________________________

Gene symbol Accession number Primer sequences

_____________________________________________________________________________

ACTB^1^ NM_001033084 GTGCGTGACATTAAGGAG (s)

AGGAAGGAAGGTTGGAAG (as)

CYP19A1 NC_000015 (NM_031226) GTTGAGAGCATACAGAAGATA (s)

TGACACTATTGGCAAGGATGG (as)

ESR1 NC_000006 (NM_000125) GCTGCTGGCTACATCATC (s)

AGGACTCGGTGGATATGG (as)

ESR2 NC_000006 (NM_001437) ATACCTTCCTCCTATGTAGA (s)

GCTGACTCTCCTTCTTCC (as)

HSD17B1 NC_000005 (NM_000413) GACCAGCAACCAGCACAG (s)

CACGCAATCTCAAGGATAAGC (as)

HSD17B2 NC_000016 (NM_002153) GAGGAATGGCGAAGAACC (s)

TAGTCAGTCATAAGAAGAAGC (as)

NR5A1 NC_000009 (NM_004959) GTTGAGAGCATACAGAAGATA (s)

TGACACTATTGGCAAGGATGG (as)

PGR NC_000011 (NM_000926) TTCCACTAACCAGACAGCAG (s)

GACCAGGCATAACTAACAAGG (as)

STAR NC_000008 (NM_000349) GCTCTCTACTCGGTTCTC (s)

TGTGATAACTGGCGATGG (as)

______________________________________________________________________________

^1^beta actin was amplified in all samples to check for the homogeneity of RNA content; s, sense; as, antisense.
