# Supplementary material for: An assessment of the multifactorial profile of steroid-metabolizing enzymes and steroid receptors in the eutopic endometrium during moderate to severe ovarian endometriosis
Source: Reprod Biol Endocrinol. 2019 Dec 26;17:111. doi: 10.1186/s12958-019-0553-0 (PMC6933937; doi:10.1186/s12958-019-0553-0)
Supplement: Supplementary file 2 — Additional file 2: Table S2. List of primary antibodies used in Western immunoblotting. [file 12958_2019_553_MOESM2_ESM.docx]

Additional file 2: Table S2 List of primary antibodies used in Western immunoblotting

_______________________________________________________________

Antigen Specification of Antibody applied

antibody ______________________

Primary Secondary^3^

(μg/mL)

**________________________________________________________________**

Aromatase^1^ Goat polyclonal IgG 1.0 1:1000

17β-HSD1^1^ Rabbit polyclonal IgG 0.4 1:2000

17β-HSD2^1^ Rabbit polyclonal IgG 0.4 1:2000

ERα^2^ Mouse monoclonal IgG 0.4 1:1000

ERβ^2^ Rabbit polyclonal IgG 1.0 1:2000

PR^2^ Mouse monoclonal IgG 0.4 1:1000

(PRA+ PRB)

SF-1^1^ Goat polyclonal IgG 1.0 1:1000

StAR^2^ Rabbit polyclonal IgG 0.4 1:2000

____________________________________________________________________

^1^Abcam, MA, USA. ^2^Santa Cruz Biotechnologies, TX, USA. ^3^dilution of neat serum.
