# Supplementary material for: An assessment of the multifactorial profile of steroid-metabolizing enzymes and steroid receptors in the eutopic endometrium during moderate to severe ovarian endometriosis
Source: Reprod Biol Endocrinol. 2019 Dec 26;17:111. doi: 10.1186/s12958-019-0553-0 (PMC6933937; doi:10.1186/s12958-019-0553-0)
Supplement: Supplementary file 3 — Additional file 3: Table S3. Sensitivity, specificity, intra- and inter-assay coefficients of variances (CV) and per cent recovery efficiency of endometrial tissue steroids estimated. [file 12958_2019_553_MOESM3_ESM.docx]

Additional file 3: Table S3 Sensitivity, specificity, intra- and inter-assay coefficients of variances (CV) and per cent recovery efficiency of endometrial tissue steroids estimated

_____________________________________________________________________________

Steroid Sensitivity Specificity: Analyte CV (%) Recovery (%)

(pg/mL) (% cross-reactivity) _____________________

Intra-assay Inter-assay

____________________________________________________________________________

Progesterone 160 17-HP (1), 11-HP (25), 1.0 4.5 100

Corticosterone (0.01),

Pregnenolone (0.9),

DOC (0.3), Deoxycortisol

(0.03), Cortisol (0.002)

Testosterone 90 5αDHT (16), 2.2 6.4 101

Androstenediol (1),

A4 (0.4), Androsterone

(<0.1), DHEA (<0.1), P4

(<0.1), E2 (<0.1), Estriol

(<0.01), Cortisol (0.01),

Pregnenolone (0.01)

Estrone 20 P4 (0.0), T (0.0), 2.3 6.5 99

E2 (2.5), Estriol (2.1)

Estradiol 14 E1 (0.2), Estriol (0.6), 2.0 6.8 102

Cortisol (0.06),

Prednisolone (0.09),

Corticosterone (<0.01),

P4 (<0.01), 17-HP

(<0.05), Pregnenolone

(<0.05), T (<0.01)

____________________________________________________________________________

DHEA, dehydroepiandrostenedione; DOC, deoxycorticosterone; E1, estrone; E2, estradiol; T, testosterone; P4, progesterone; HP, hydroxyprogesterone.
