# Supplementary material for: An assessment of the multifactorial profile of steroid-metabolizing enzymes and steroid receptors in the eutopic endometrium during moderate to severe ovarian endometriosis
Source: Reprod Biol Endocrinol. 2019 Dec 26;17:111. doi: 10.1186/s12958-019-0553-0 (PMC6933937; doi:10.1186/s12958-019-0553-0)
Supplement: Supplementary file 4 — Additional file 4: Table S4. Endometrial transcript and protein expression of factors involved in steroid biosynthesis in patients with and without endometriosis. [file 12958_2019_553_MOESM4_ESM.docx]

Additional file 4: Table S4 Endometrial transcript^1^ and protein^2^ expression of factors involved in steroid biosynthesis in patients with and without endometriosis

___________________________________________________________________________________________________________

Group 1 (Control) 2 (Ovarian endometriosis) *P value**

_________ ______________________________________ _______________________________________

Fertility F IF F IF

status

______________________________________________________________________________________________

Menstrual P, ***n*** S, ***n*** P, ***n*** S, ***n*** P, ***n*** S, ***n*** P, ***n*** S, ***n***

phase

_______________________________________________________________________________________________

Parameter Expression value in median (ranges)

__________________________________________________________________________________________________________

*NR5A1* 6.0 5.4 5.3 6.4 5.0 5.0 3.9 3.4 *0.61*

(5.3-7.6) (5.1-6.4) (5.2-5.4) (5.6-7.9) (0.6-11.7) (1.0-17.7) (1.1-4.6) (0.0-5.6)

***10 4 4 4 11 7 6 9***

[SF-1] 0.6 0.7 0.4 0.8 1.5 1.7 0.2 0.6 *0.48*

(0.4-0.8) (0.5-0.9) (0.4-0.7) (0.5-0.8) (0.2-3.5) (0.5-3.0) (0.0-0.2) (0.1-10.0)

***4 4 4 4 7 4 7 5***

*STAR* 6.5 7.2 7.0 6.5 3.6 7.5 0.8 1.4 *0.10*

(3.4-7.6) (6.5-7.6) (6.5-7.5) (2.7-7.5) (2.2-6.8) (2.2-10.0) (0.4-3.1) (0.6-1.9)

***10 4 4 4 11 7 6 9***

[StAR] 9.0 8.1 12.5 7.7 14.7 0.6 0.8 2.6 *0.58*

(8.7-9.2) (7.7-8.8) (10.3-14.9) (7.5-8.0) (0.4-28.2) (0.3-3.9) (0.1-9.4) (0.1-91.6)

***4 4 4 4 7 4 7 5***

*CYP19A1* 6.1 3.3 2.9 3.5 5.7 2.7 1.6 1.2 *0.58*

(3.0-7.5) (2.0-4.5) (2.3-3.5) (2.5-4.1) (1.8-18.8) (0.7-5.5) (0.0-2.8) (0.0-2.3)

***10 4 4 4 11 7 6 9***

[Aromatase] 10.0^a^ 5.3 13.7 7.7 5.1 1.0 0.4^b^ 2.0 *0.01*

(9.9-10.5) (4.7-5.8) (11.0-14.4) (6.1-8.3) (0.7-8.9) (0.0-2.0) (0.2-0.9) (1.4-4.5)

***4 4 4 4 7 4 7 5***

*HSD17B1* 6.6 7.7 6.7 5.7 7.5 7.3 6.1 5.7 *0.06*

(5.4-9.2) (5.9-8.9) (4.5-8.4) (4.9-8.2) (5.4-9.2) (5.0-8.9) (4.6-9.0) (5.3-8.7)

***10 4 4 4 11 7 6 9***

[17β-HSD1] 0.6 0.2 0.7 0.4^c^ 18.1 5.5 8.0 79.2 *0.01*

(0.4-0.8) (0.1-0.2) (0.6-0.8) (0.2-0.4) (4.3-44.1) (0.4-28.0) (0.1-27.1) (45.7-202.4)

***4 4 4 4 7 4 7 5***

*HSD17B2* 6.0 5.8 4.6 5.1 6.7 6.4 6.4 5.4 *0.11*

(5.5-6.5) (5.1-6.0) (4.0-5.0) (2.6-6.2) (4.9-7.9) (5.0-7.2) (2.7-7.1) (4.4-7.6)

***10 4 4 4 11 7 6 9***

[17β-HSD2] 1.0 0.9 1.0 0.9 3.6 0.2 0.6 1.1 *0.21*

(1.0-1.1) (0.8-0.9) (0.8-1.0) (0.9-0.9) (0.0-37.2) (0.2-1.5) (0.3-0.8) (0.9-5.8)

***4 4 4 4 7 4 7 5***

___________________________________________________________________________________________________________

*computed from Kruskal-Wallis test. ^1^log base 2 of transcript copy number obtained using GAPDH as standard in qRTPCR, shown in *italics.* ^2^integrated optical density normalized to total loading protein (25 μg Bradford protein) in WB, shown in *square brackets.* F, fertile; IF, infertile; P, proliferative phase; S, secretory phase. ^a^P< 0.05 in comparisons between fertile proliferative phase samples of group 1 (control) as compared to fertile proliferative phase endometrial samples of group 2 (OE). ^b^P< 0.05 in comparisons between infertile secretory and infertile proliferative group 2 (OE) samples. ^c^P< 0.05 in comparisons between infertile secretory phase samples of group 1 (control) and group 2 (OE) samples.
