# Supplementary material for: An assessment of the multifactorial profile of steroid-metabolizing enzymes and steroid receptors in the eutopic endometrium during moderate to severe ovarian endometriosis
Source: Reprod Biol Endocrinol. 2019 Dec 26;17:111. doi: 10.1186/s12958-019-0553-0 (PMC6933937; doi:10.1186/s12958-019-0553-0)
Supplement: Supplementary file 5 — Additional file 5: Table S5. Endometrial transcript and protein expression of estrogen and progesterone receptors in patients with and without endometriosis. [file 12958_2019_553_MOESM5_ESM.docx]

Additional file 5: Table S5 Endometrial transcript^1^ and protein^2^ expression of estrogen and progesterone receptors in patients with and without endometriosis

___________________________________________________________________________________________________________

Group 1 (Control) 2 (Ovarian endometriosis) *P value**

______________________________________________________________________________________________

Fertility F IF F IF

status

________ ________________ ________________ ____________________ _________________

Menstrual P, ***n*** S, ***n*** P, ***n*** S, ***n*** P, ***n*** S, ***n*** P, ***n*** S, ***n***

phase

__________________________________________________________________________________________________

Parameter Expression value in median (ranges)

__________________________________________________________________________________________________________

*ESR1* 6.9 6.4 7.3 7.4 3.9 4.6 0.8 7.3 *0.21*

(1.1-8.3) (6.0-11.1) (6.9-7.6) (0.7-8.1) (1.6-14.9) (3.4-13.0) (0.3-4.2) (0.9-17.5)

***10 4 4 4 11 7 6 9***

[ERα] 3.7 2.7 4.6 2.0 4.3 1.3 0.5 1.6 *0.91*

(3.2-4.0) (2.2-3.3) (4.2-4.6) (1.9-2.8) (0.2-24.6) (0.4-2.1) (0.3-8.7) (1.2-4.5)

***4 4 4 4 7 4 7 5***

*ESR2* 5.5 6.0 3.5 6.5 5.6 4.9 0.7 2.2 *0.18*

(3.6-6.1) (5.0-6.3) (2.3-4.8) (5.8-6.9) (1.8-9.0) (2.2-16.8) (0.0-2.4) (0.0-3.4)

***10 4 4 4 11 7 6 9***

[ERβ] 7.2 5.4 10.9 6.7 1.8 0.5 0.6 2.3 *0.21*

(5.4-8.5) (1.4-6.5) (9.9-12.0) (2.1-7.9) (0.6-8.0) (0.3-0.6) (0.0-1.0) (0.0-15.4)

***4 4 4 4 7 4 7 5***

*PGR* 3.2 3.5 2.7 2.7 15.1 15.7 1.1^a^ 2.0 *0.009*

(2.1-6.8) (2.9-6.7) (2.4-2.9) (2.4-5.0) (11.2-23.2) (12.1-20.8) (0.2-6.3) (0.1-5.4)

***10 4 4 4 11 7 6 9***

[PRA] 0.6 1.1 0.7 0.5^b^ 9.5 3.8 1.2 4.1 *0.02*

(0.5-0.7) (1.0-1.3) (0.7-0.8) (0.4-0.9) (1.3-16.1) (1.5-6.7) (0.5-2.8) (1.5-12.0)

***4 4 4 4 7 4 7 5***

[PRB] 8.8 9.0 5.1 6.8^c^ 3.9 1.2 2.7 1.2 *0.04*

(7.1-9.7) (1.9-9.1) (2.9-5.7) (6.4-6.8) (1.1-12.0) (1.0-1.3) (0.7-10.0) (0.7-1.9)

***4 4 4 4 7 4 7 5***

____________________________________________________________________________________________________________

*computed from Kruskal-Wallis test. ^1^log base 2 of transcript copy number obtained using GAPDH as standard in qRTPCR, shown in *italics.* ^2^integrated optical density normalized to total loading protein (25 μg Bradford protein) in WB, shown in *square brackets.* F, fertile; IF, infertile; P, proliferative phase; S, secretory phase. ^a^P< 0.01 in comparisons between infertile proliferative phase and infertile secretory phase OE (group 2) samples. ^b^P< 0.05, ^c^P< 0.01 in comparisons between infertile secretory phase samples of control (group 1) and OE (group 2).
