# Supplementary material for: An assessment of the multifactorial profile of steroid-metabolizing enzymes and steroid receptors in the eutopic endometrium during moderate to severe ovarian endometriosis
Source: Reprod Biol Endocrinol. 2019 Dec 26;17:111. doi: 10.1186/s12958-019-0553-0 (PMC6933937; doi:10.1186/s12958-019-0553-0)
Supplement: Supplementary file 6 — Additional file 6: Table S6. Tissue steroid hormone concentration (pmol/mg protein) in endometrium of patients with and without endometriosis. [file 12958_2019_553_MOESM6_ESM.docx]

Additional file 6: Table S6 Tissue steroid hormone concentration (pmol/mg protein) in endometrium of patients with and without endometriosis

___________________________________________________________________________________________________________

Group 1 (Control) 2 (Ovarian Endometriosis) ***P value****

_____________________________________________________________________________________________________________

Fertility F IF F IF

status _________________ ________________ ____________________ ___________________

Menstrual P^1^ S^1^ P^1^ S^1^ P^1^ S^1^ P^1^ S^1^

phase

__________________________________________________________________________________________________________

Parameter Expression value in median (ranges)

__________________________________________________________________________________________________________

Progesterone 102.7 61.6 358.4 175.0 168.96 165.1 108.8 302.1 ***0.91***

(P4) (87.7-139.5) (85.4-225.6) (197.1-382.4) (135.7-334.1) (102.1-185.3) (102.1-350.4) (57.0-112.6) (207.4-418.6)

Testosterone 36.4 71.8 157.2 61.6 80.15 69.0 18.9 153.3 ***0.48***

(T) (27.3-47.6) (31.9-72.5) (79.8-163.5) (46.9-144.2) (47.6-86.1) (50.4-161.4) (16.8-42.0) (75.6-167.3)

Estradiol 24.4 27.8 87.0 23.7 30.7 32.9^a^ 20.0 92.5 ***0.01***

(E2) (13.3-32.9) (6.7-42.2) (77.3-119.1) (23.7-69.6) (14.8-46.6) (23.3-88.8) (11.1-50.7) (83.3-159.1)

Estrone 7.4 14.4 30.7 12.6 13.7 12.2 4.4 24.4 ***0.21***

(E1) (5.2-9.3) (8.1-18.9) (15.5-40.3) (11.5-28.9) (5.6-16.3) (10.4-37.0) (3.3-8.1) (12.2-27.8)

____________________________________________________________________________________________________________

*computed from Kruskal-Wallis test. ^1^n = 4. F, fertile; IF, infertile; P, proliferative phase; S, secretory phase. ^a^P< 0.05 in comparisons between secretory phase samples of infertile and fertile sub-groups of group 2 (OE) samples.
