# Supplementary material for: An assessment of the multifactorial profile of steroid-metabolizing enzymes and steroid receptors in the eutopic endometrium during moderate to severe ovarian endometriosis
Source: Reprod Biol Endocrinol. 2019 Dec 26;17:111. doi: 10.1186/s12958-019-0553-0 (PMC6933937; doi:10.1186/s12958-019-0553-0)
Supplement: Supplementary file 7 — Additional file 7: Table S7. Correlation between transcripts and protein levels. [file 12958_2019_553_MOESM7_ESM.docx]

Additional file 7: Table S7 Correlation between transcripts and protein levels

___________________________________________________________

Parameter R-value *P value*

Gene transcript *vs.*

corresponding protein

___________________________________________________________

CYP19A1 *vs.* aromatase 0.52 0.37

ESR1 *vs*. ERα 0.90 0.04

ESR2 *vs.* ERβ 0.29 0.64

HSD17B1 *vs.* 17β-HSD1 -0.37 0.54

HSD17B2 *vs.* 17β-HSD2 -0.67 0.20

NR5A1 *vs.* SF-1 0.35 0.56

PGR *vs.* PRA 0.72 0.17

PGR *vs.* PRB -0.65 0.24

STAR *vs.* StAR 0.94 0.02

_____________________________________________________________
