# Supplementary figures and images for: An assessment of the multifactorial profile of steroid-metabolizing enzymes and steroid receptors in the eutopic endometrium during moderate to severe ovarian endometriosis
Source: Reprod Biol Endocrinol. 2019 Dec 26;17:111. doi: 10.1186/s12958-019-0553-0 (PMC6933937; doi:10.1186/s12958-019-0553-0)

Additional file 8: Figure S1


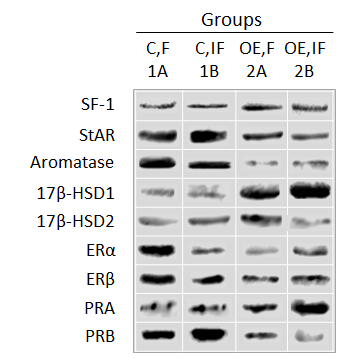

Supplement: Supplementary file 8 — Additional file 8: Figure S1. Representative images of Western blots showing the effect of the fertility history on the levels of the SF-1, StAR, aromatase, 17β-HSD1, 17β-HSD2, ERα, ERβ, PRA and PRB proteins in endometrial samples from the control, fertile (C,F; group 1A); control, infertile (C,IF; group 1B); OE, fertile (OE,F; group 2A); and OE, infertile (OE,IF; group 2B) groups. Tissue lysates of samples from these groups (25 μg of proteins, concentrations were determined by using the Bradford assay) were subjected to electrophoretic separation followed by immunoblot analysis. The relative optical densities were measured by performing an integrated image analysis and normalizing the value to the μg of total protein. [file 12958_2019_553_MOESM8_ESM.docx]

Additional file 9: Figure S2


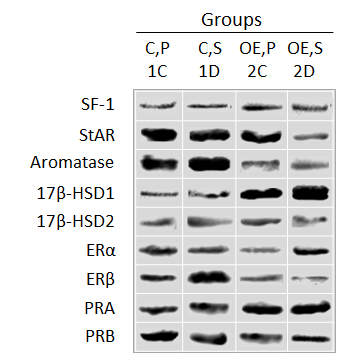

Supplement: Supplementary file 9 — Additional file 9: Figure S2. Representative images of Western blots showing the effect of the menstrual cycle phase on the levels of the SF-1, StAR, aromatase, 17β-HSD1, 17β-HSD2, ERα, Erβ, PRA and PRB proteins in endometrial samples from the control, proliferative (C,P; group 1C); control, secretory (C,S; group 1D); OE, proliferative (OE,P; group 2C); and OE, secretory (OE,S; group 2D) groups. Tissue lysates of samples from these groups (25 μg of protein, concentrations were determined by using the Bradford assay) were subjected to electrophoretic separation followed by immunoblot analysis. The relative optical densities were measured by performing an integrated image analysis and normalizing the values to the μg of total protein. [file 12958_2019_553_MOESM9_ESM.docx]
